# The Leptin System and Diet: A Mini Review of the Current Evidence

**DOI:** 10.3389/fendo.2021.749050

**Published:** 2021-11-24

**Authors:** Kenny Mendoza-Herrera, Andrea A. Florio, Maggie Moore, Abrania Marrero, Martha Tamez, Shilpa N. Bhupathiraju, Josiemer Mattei

**Affiliations:** Department of Nutrition, Harvard TH Chan School of Public Health, Boston, MA, United States

**Keywords:** leptin, diet, obesity, leptin resistance, dietary interventions

## Abstract

Leptin promotes satiety and modulates energy balance and weight. Diet-induced obesity leads to leptin resistance, exacerbating overeating. We reviewed the literature on the relationship between diet and leptin, which suggests that addressing leptin resistance through dietary interventions can contribute counteracting obesity. Albeit some limitations (e.g., limited rigor, small samples sizes), studies in animals and humans show that diets high in fat, carbohydrates, fructose, and sucrose, and low in protein are drivers of leptin resistance. Despite methodological heterogeneity pertaining to this body of literature, experimental studies show that energy-restricted diets can reduce leptinemia both in the short and long term and potentially reverse leptin resistance in humans. We also discuss limitations of this evidence, future lines of research, and implications for clinical and public health translations. Main limitations include the lack of a single universally-accepted definition of leptin resistance, and of adequate ways to accurately measure it in humans. The use of leptin sensitizers (drugs) and genetically individualized diets are alternatives against leptin resistance that should be further researched in humans. The tested very-low-energy intervention diets are challenging to translate into wide clinical or population recommendations. In conclusion, the link between nutritional components and leptin resistance, as well as research indicating that this condition is reversible, emphasizes the potential of diet to recover sensitivity to this hormone. A harmonized definition of leptin resistance, reliable methods to measure it, and large-scale, translational, clinical, and precision nutrition research involving rigorous methods are needed to benefit populations through these approaches.

## Introduction

Discovered in 1994, leptin is an adipokine, a protein that functions as a hormone (1). Two major producers and secretors of leptin are the adipose tissue and the gastric mucosa (1–4). Leptin promotes satiety and has a central role in energy balance and weight management. A primary leptin signaling pathway is its interaction with the hypothalamus, which modulates the central nervous system to regulate metabolic homeostasis. Leptin also interacts with insulin, impacting glucose and lipid homeostasis (5). In normal physiological conditions, serum leptin increases in the fed state, decreases in the fasted state, and is directly correlated with total body fat mass: if there is more adipose tissue, there is more leptin, thereby reflecting long-term energy availability (6). Furthermore, in conjunction with its long receptor expressed by most immune cells, leptin has proinflammatory activity (1).

The role of leptin in diet-related disease has been well-studied. This mini review summarizes a significant body of literature documenting the interconnection between leptin and food intake and examines the evidence around the paradoxical phenomenon known as leptin resistance (LR), whereby chronically elevated leptin levels lead to an inefficient transport of leptin into the brain, reduced hypothalamic leptin receptor (LepRb) expression, alteration of leptin signaling cascade in the hypothalamus, among other mechanisms that impair leptin function over time (7, 8). With research pointing toward leptin sensitization as a promising weight loss intervention, we primarily discuss nutritional interventions aimed at normalizing leptin levels and sensitivity. Gaps and limitations in the current evidence, as well as areas for future research and clinical translation, are also discussed.

## Leptin Physiology

Leptin circulates in proportion to body energy stores and food availability (9). When energy reserves are high, leptin is secreted by adipocytes into the bloodstream (constitutive secretion). Gastric leptin secretion (regulated) rapidly responds to the stimuli caused by food intake. Being this hormone resistant to proteolysis in the intestinal tract, gastric leptin contributes with a large part of the circulating leptin levels, but especially with those occurring at food ingestion and digestion (2–4).

Once in the bloodstream, leptin crosses the blood–brain barrier to bind its receptor in the hypothalamus to signal satiety and decrease hunger and food intake (8, 10, 11). Aside from the central action of leptin described above, this hormone has peripheral actions occurring in different tissues (12, 13). For example, interacting with cholecystokinin in the stomach, leptin increases vagal afferent activity controlling the gastric emptying which in turn contributes to satiety (14). At intestinal level in postprandial conditions, leptin boosts carbohydrate and protein absorption through an increased expression of glucose transporters (GLUT-2, GLUT-5, and sodium-glucose cotransporter-1) (15–17) and the activation of the proton-dependent di- and tri-peptide transporter PepT1 (18). Conversely, leptin diminishes lipid release into the bloodstream after downregulating apolipoproteins (19). Another important function of leptin is to reduce ectopic accumulation of fat by stimulating fatty acid oxidation in the liver and muscular tissue (20–22).

When adipose reserves or food availability are low, leptin levels decrease, thereby increasing food intake to ensure a steady supply of metabolic fuel (8). It is reasonable to think that leptin decrements to increase food intake produce a stronger signal than the one produced by a rise of this hormone to reduce food intake as a survival function resulting from the long-term evolutionary challenges dominated by food shortage (7, 23). Along with the rationale above, the correlation between leptin and body weight suggests that leptin signaling evolved as an indicator of high energy reserves and a lessened need for food intake. However, the current body of evidence paints a more complex picture.

## Leptin Resistance and Diet-Induced Obesity

Diet-induced obesity (DIO) interferes with the hormonal regulation of body weight and hunger (24, 25), as leptin resistance and hyperleptinemia frequently co-exist. Individuals with mutations in the leptin gene, regardless of their obesity status, have been treated with exogenous sources of leptin to restore normal function. It was suggested that exogenous sources of leptin could also be used in individuals with obesity, who were thought to lack sufficient leptin (26). However, this proved to be ineffective (27). Recent research clarified that individuals with DIO tend to have chronic hyperleptinemia: elevated, rather than reduced, circulating leptin for their adipose mass (28). Animal models suggest that when circulating leptin levels are chronically elevated, hypothalamic leptin receptors become desensitized, akin to insulin resistance seen in type 2 diabetes. In LR, the hypothalamus becomes increasingly less responsive to leptin, hunger remains elevated, and food intake does not decrease although energy in the form of adipose is abundant (28). Multiple mechanisms compromising the signaling cascade and action of leptin and its receptor in obesity conditions have been described, although specific pathways remain unclear (8).

## Measuring Leptin Resistance

LR cannot yet be consistently measured (29). Currently, some methods such as the Free Leptin Index (29), a mathematical approach to the diagnosis of LR (30), or several proxy variables of this condition are used in animal models and human studies. However, there is no consensus criteria for a diagnosis. Studies in animals utilize invasive techniques that cannot be ethically or practically performed in human studies, such as intracerebroventricular and intraperitoneal injection of leptin, as well as microvessel isolation. With these methods, investigators have been able to directly evaluate molecular indicators of leptin signaling cascade and behavior regarding responsiveness to this hormone, including changes in food intake (31, 32). Leptinemia is the most frequent proxy variable of LR used in human studies, which is commonly complemented with other surrogate indicators of leptin action, including appetite and satiety scores, or food intake and changes in body weight also used in animal studies. Furthermore, fundic mucosa levels of leptin mRNA in humans and leptin content in gastric juice of mice have been used to measure gastric leptin response to food intake (17, 33). A more precise definition of LR requires a better understanding of the “multiple molecular, neural, behavioral, and environmental mechanisms” (34) that underlie the body’s response to chronic hyperleptinemia and its indicators.

## Circulating Leptin Levels, Leptin Resistance, and Macronutrients

Dietary factors such as overeating and critical nutrients, including fat or sugars, can trigger molecular mechanisms giving rise to LR (35). For example, dietary sugar and saturated fats elevate plasma triglycerides, which in turn may trigger the onset of LR by inducing resistance to leptin transport at the blood-brain barrier (36). Despite heterogeneity in methods to evaluate LR and its association with diet, several animal and human studies demonstrate how particular macronutrient patterns correlate to circulating leptin levels and other indicators of leptin desensitization. Furthermore, studies have shown that gastric leptin secretion also responds to the intake of certain macronutrients, thereby activating mechanisms that may lead to peripheral LR and be critical early points in the pathophysiology of obesity. This evidence considers the relevant role of gastric leptin in the delivery of macronutrients from the intestine into the bloodstream and the regulation of food intake (37). The body of literature mentioned above is shown in **Table 1** and described in the following sections.

**Table 1:** Summary of studies linking nutritional components to leptin resistance.

Study	Type of Study	Study Characteristics	Diet Characteristics and Relevant Study Procedures	Main Results^a^
**Fat**				
**El-Haschimi K, et al. (** 31 **)**	Mouse	- 44 male mice (4 weeks old).	- Group 1: HFD, with 45% energy from fat. - Group 2: LFD, with 10% energy from fat. - Evaluations carried out at weeks 4 and 15. - The ability of leptin to induce STAT3 (signal transducer and activator of transcription) signaling was evaluated in both groups.	- Compared with those fed the LFD, mice fed the HFD weighed 24% more after 15 weeks. - The HFD was associated with higher leptin levels. - After 15 weeks, the same dose of intraperitoneal leptin that was effective in mice fed the LFD was unable to induce STAT3 DNA binding activity in hypothalamus of mice of Group 1, indicating leptin resistance. - Similar doses of intracerebroventricular induced STAT3 activation in mice with 15 weeks of diet-induced obesity, which suggests a failure to deliver leptin to areas of action in the hypothalamus.
**Lin L, et al. (** 32 **)**	Rat	- 50 male rats (initial weight of 250 ± 2 g).	- Group 1: HFD, with 56% energy from fat, 4.78 kcal/g. - Group 2: LFD, with 10% energy from fat, 3.66 kcal/g. - Protein in both diets: with 24% energy from protein. - Duration: minimum of 2 weeks. - An injection of leptin (0.5mg/kg) was given intraperitoneally to test changes in food intake in both groups.	- Leptin reduced food intake in rats eating the LFD (P < 0.0068). - Food intake did not diminish after leptin administration in the rats eating the HFD. - Rats eating the HFD had significantly higher body weight (HFD: 483 ± 13.1 g *vs*. LFD: 437 ± 6.3 g) and serum leptin levels at the end of the study. - Rats fed the LFD had higher hypothalamic NPY mRNA levels than those fed the HFD (P <0.0015). - Hypothalamic 5-HT_2C_ receptor mRNA levels were lower in rats fed the HFD (P <0.0003).
**Kratz M, et al. (** 38 **)**	Human	- 55 participants (30 men age of 28.9 ± 5.2 y, 25 women, age of 22.8 ± 4.2 y) without obesity (BMI <27 kg.m^2^).	- All participants consumed a HFD (high in saturated fatty acids) for 2 weeks. - Afterwards, participants were randomly assigned to 1 of 3 HFDs differing in fatty acid composition (4 weeks). - Group 1: refined olive oil (high monounsaturated fatty acids; 10 men, 9 women). - Group 2: sunflower oil (high in n-6-polyunsaturated fatty acids; 10 men, 9 women). - Group 3: rapeseed oil (high in monounsaturated fatty acids and α-linolenic acid (18:3n-3; 10 men, 7 women).	- The rapeseed oil diet: leptin levels increased slightly in men (0.25 ng/ml, P=0.021), but decreased in women (4.70 ng/ml, P=0.002). - Diets high in both the olive and sunflower oils did not affect leptin concentrations. - The study suggest that leptin levels were affected by the high amount of α-linolenic acid.
**Le Beyec J, et al. (** 33 **)**	Mouse	- 8 mice (6–8 weeks old).	- Group 1: standard diet, with 3% energy from fat, 48% from carbohydrates, and 16% protein. - Group 2: HFD, with 45% energy from fat, 35% from carbohydrates, and 20% from protein. - Gastric leptin levels measured weekly.	- Compared with those fed the standard chow, mice fed the HFD had a significant 50% increase in gastric leptin levels after 12 weeks. - Adipocyte-derived plasma leptin levels among mice fed the HFD significantly increased twofold after 12 weeks, though leptin levels had significantly increased after only 4 weeks of the HFD.
**Carbohydrates**				
**Dirlewanger M, et al. (** 39 **)**	Human	- 10 women without obesity (age 22.4 y (range: 20-26), with BMI 21.9 ± 2.2 kg.m^2^).	- 3 periods of 3 days. - 1st: an isoenergetic diet with 50% energy from carbohydrates, 35% lipid, and 15% protein. - 2nd: hyperenergetic diet with 40% energy excess from carbohydrates (bread, rice, biscuits, and sugar). - 3rd: hyperenergetic diet with 40% energy excess from fat.	- Compared to the isoenergetic diet, carbohydrate overfeeding led to a 28% increase in leptin levels (postabsorptive state). - Fat overfeeding had no effect on leptin concentration as compared with the isoenergetic diet. - Twenty-four-hour energy expenditure increased by 7% in carbohydrate overfeeding as compared with the isoenergetic diet (P < 0.05). - Fat overfeeding had no effect on energy expenditure.
**Romon M, et al. (** 40 **)**	Human	- 11 men (age 23.9 ± 3.2 y, BMI 22.3 ± 1.8 kg.m^2^) and 11 women (age 21.5 ± 1.9 y, BMI 21.6 ± 1.8 kg.m^2^) without obesity.	- Two isoenergetic meals with different amounts of carbohydrates (81% of total energy, 90 g of maltose solution plus other foods and supplements) and fat (79%), with similar protein content (~18%).	- In women and men, leptin concentrations were higher 4-9 h after the carbohydrate meal than the fat meal and during fast. - In women, leptin levels were also higher 5-9 h after the fat meal than during the fasting experiment. - No statistically significant differences in hunger and satiety scores between carbohydrate and fat meals in women (between 1 and 9 h postprandially). - In men, satiety scores were higher 3 and 4 h after the fat meal than the carbohydrate meal, hunger scores were lower 6 and 7 h after the high-fat meal than the carbohydrate meal.
**Fructose**				
**Shapiro A, et al. (** 41 **)**	Rat	- 24 male rats (age 3 months, body weight 459 ± 17.2 g).	- 1 week: chow (with 0% energy from sugar (sucrose), 58% carbohydrates other than sugar [starch/fiber], 17% fat, 25% protein, 3.1 kcal/g). - Afterwards, 14 rats were switched to a high-fructose/high-fat diet (with 40% energy from fructose, 10% carbohydrates other than fructose [starch/fiber], 30% fat, 20% protein) and 10 rats received a sugar-free/HFD (with50% energy from carbohydrates other than sugar [starch/fiber], 30% fat, 20% protein) ad libitum. - On day 70, 8 rats in the high-fructose/high-fat group were switched to the sugar-free/HFD.	- At day 65: after peripherally administered leptin (0.6 mg/kg), rats on the sugar-free/HFD significantly reduced food intake; those on the high-fructose/HFD did not decrease food intake (indicator of LR). - Changing from the high-fructose/HFD to the sugar-free/HFD: 10% reduction of cumulative food intake over the 5-week period (*P* = 0.04). -Removal of fructose from the high-fructose/HFD improved leptin responsiveness in leptin resistant rats: reduction in food intake after leptin administration 18 days after the switch to the sugar-free/HFD (P = 0.03).
**Le KA, et al. (** 42 **)**	Human	- 7 healthy men (age 24.7 ± 1.3 y, BMI 19-25 kg.m^2^)	- During initial 2 weeks: isoenergetic diet with fructose intake <20 g/d (with55% energy from carbohydrates, 30% fat, and 15% protein). Sucrose-sweetened or artificially sweetened drinks and food were included. - Subsequent 4 weeks: high-fructose diet (excess of 18% of the participants’ daily energy requirement).	- The high-fructose diet was associated with a significant increase in fasting leptin concentrations (48%, *P <*0.05) after 1 week.
**Sakar Y, et al. (** 17 **)**	Mouse	- 48 male mice (6-8 weeks old).	- Mice were first fasted for 18 hours, then orally fed 1 of 4 treatments below. - Group 1: control group, fed an oral saline. - Group 2: fructose (2 g/kg). - Group 3: leptin (3 ng/kg). - Group 4: fructose and leptin.	- Compared to control group mice, mice fed the fructose solution had a significant 10-fold increase in basal leptin content in gastric juice (observed 15 minutes after administration). - Plasma leptin levels did not significantly change 15 minutes after oral administration, though levels did significantly increase 4 hours after oral fructose.
**Sucrose**				
**Harris RB and Apolzan JW (** 43 **)**	Rat	- 60 male rats.	- Group 1: chow (with 24% energy from protein [minimum], 4% fat [minimum]). - Group 2: free access to chow and lard. - Group 3: free access to chow and 30% sucrose solution. - Group 4: choice diet (free access to chow, lard, and 30% sucrose solution). - Group 5: LFD. - Group 6: diet with 60% energy from fat.	- Day 17 and 20: food intake was inhibited by intraperitoneally injected leptin (2 mg/kg) in all groups except in groups 3 and 4 after 14 and 36 hours. - Day 23: serum leptin concentrations were higher in Groups 4 (17 ± 3 ng/ml) and 6 (15 ± 2 ng/ml) than in the rest of the groups (6-12 ng/ml).
**Protein**				
**de França SA, et al. (** 44 **)**	Rat	- 12 male rats (100 g, age 30 days)	- Duration: 15 days. - Controls (n = 6): diet with 17% energy from protein. - Intervention group (n = 6): low-protein diet, with 6% energy from protein.	- Food consumption of the intervention group was 14% higher than that of the control group. - The low-protein diet increased leptin concentrations by 100% (P < 0.01).
**Du F, et al. (** 45 **)**	Rat	- 63 male rats (150 g).	- Six groups: isocaloric diets with 2%, 5%, 8%, 10%, 15%, and 20% energy from protein.	- Food intake showed a quasi-bell-shaped response curve over the range of the protein content (with its peak in rats fed 8–10% energy from protein). - Food intake was significantly reduced in rats fed a diet with 2% energy from protein. - Body fat steadily increased between the 15% and the 8% energy from protein groups, and sharply declined between the 5% and 2% energy from protein groups. - Leptin levels were higher in rats fed the 5 and 8% energy from protein diets than in rats fed 20% protein.
**Weigle DS, et al. (** 46 **)**	Human	- 19 healthy adults (3 men, 16 women). - Age: 41 ± 11 y (range: 27–62). - BMI: 26.2 ± 2.1 kg.m^2^ (22.5–30.1)	- 3 diets were subsequently tested. - Diet 1: weight-maintaining diet (with 15% energy from protein, 35% fat, and 50% carbohydrates) for 2 weeks. Diet 2: isocaloric high-protein diet (with 30% energy from protein, 20% fat, and 50% carbohydrates) for 2 weeks. - Diet 3: an ad libitum diet (with30% energy from protein, 20% fat, and 50% carbohydrates) for 12 weeks.	- Satiety significantly increased with the isocaloric high-protein diet, though the AUC for leptin did not change. - Decrements associated with the ad libitum, high-protein diet along with a significantly decreased leptin AUC: spontaneous energy intake (441 ± 63 kcal/d), body weight (4.9 ± 0.5 kg), and fat mass 3.7 ± 0.4 kg.

^a^Statistical measures presented in this table are heterogeneous since they are shown as they were available in the reviewed studies.

BMI, body mass index; AUC, area under the plasma concentration versus time curve; HFD, high-fat diet; LFD, low-fat diet.

### Dietary Fat

High-fat diets have been widely used to establish DIO and LR models in animal studies (32). Both hyperleptinemia (47) and hypothalamic inflammation (48) have been proposed as causal mechanisms for the impact of dietary fat on LR development, and other studies show the complexity behind these phenomena.

For example, compared with rodents fed low-fat diets, mice made obese by 15-week feeding of high-fat diet (10% *vs*. 45% fat) experienced increases in weight and in plasma leptin levels. Compared to those fed the low-fat diet, mice fed the high-fat diet also became resistant to exogenous leptin administered to the peritoneum [decreased activation of hypothalamic signal transducer and activator of transcription 3 (STAT3)]. Despite the increased plasma leptin levels and body weight before this period, LR was significant only until week 15. While intracerebroventricular leptin induced hypothalamic signaling in mice fed the high-fat diet in this study in week 15, this induction was 75% lower than that in mice fed the low-fat diet (31). Based on these results, the authors (31) suggest that LR derived from a high-fat diet progresses with the course of the diet and may be partially explained by two mechanisms: irregularities in the access to hypothalamic action sites that hamper the activation of hypothalamic signaling by peripheral leptin and intracellular signaling abnormalities in leptin-responsive neurons in the hypothalamus found upstream of the STAT3 activation. Interestingly, another animal study showed that resistance to peripheral leptin could be induced within a 2-week exposure period to high-fat diet (56% fat, 20% corn starch *vs*. 10% fat, 66% corn starch) even before increases in leptin levels and body weight occur (32).

In animals and humans, several studies have found only certain types of dietary fat to be associated with leptin levels. Kratz et al. measured serum leptin levels among 30 men and 25 women with a healthy body mass index (BMI, 18.5-24.9 kg.m^2^) after a high-fat diet intervention and found that leptin levels were only associated with the diet rich in alpha-linolenic acid (omega-3 in rapeseed oil); leptin significantly decreased in women and slightly increased in men (38). This result may be due in part to differential concentration and hormonal activity of leptin seen in males and females (24). The intervention diets rich in monounsaturated fatty acids and omega-6-polyunsaturated fatty acids did not affect leptin concentrations (38). Further, a review on the effect of fatty acid intake on obesity concluded that increased omega-6 fatty acid intake increased LR, insulin resistance, and obesity. In contrast, increased omega-3 fatty acid intake led to homeostasis and weight loss in both humans and rodents (49). Furthermore, a study in animals showed that, compared to gavage with polyunsaturated triglycerides (fish oil), administration of minimal amounts of saturated triglycerides (diluted cream) to the diet of normal-weight rats consuming a low-fat diet could induce resistance to injected leptin and elevate feeding in response to a high-fat diet, both acutely and in the long term (35).

Some have hypothesized that increased gastric leptin levels linked to the diet could be an early marker of peripheral LR. Le Beyec and collaborators studied the expression of gastric leptin and plasma leptin levels in response to a high-fat diet in mice for 12 weeks (33). The animals were fed standard chow (3% fat, 48% carbohydrates [mainly starch], and 16% protein) or a high-fat diet (45% fat, 35% carbohydrates, and 20% protein). Compared with mice fed a regular diet, those fed a high-fat diet had significantly increased gastric leptin levels (+70%; P<0.001) after one week. The gastric leptin expression in mice fed a high-fat diet experienced a significant 50% increase up to 12 weeks compared with that of mice fed a regular diet. Interestingly, the plasma leptin levels derived from adipocytes and the fat mass of mice increased only after four weeks of the high-fat diet. The authors in this study suggest that a chronic increase of gastric leptin may eventually lead to intestinal leptin resistance, similar to the peripheral leptin resistance in the vagus afferent neurons previously observed in rats with DIO (33, 50).

### Carbohydrates

Existing evidence on the association between carbohydrates and leptin level concentrations is inconclusive. In a study of healthy women, an increase in carbohydrate consumption (40% excess energy as carbohydrates derived from bread, rice, biscuit, and sugar) resulted in plasma leptin levels increasing by 28% and an increase in 24-hour energy expenditure of 7%. Overfeeding of dietary fat had no significant relationship to leptin levels or energy expenditure (39). Similarly, a feeding trial of 22 healthy participants showed a higher leptin response after a carbohydrate-rich meal (81%, 90 g of maltose solution plus other foods and supplements) compared to an isoenergetic fat-rich meal (79%) or after fasting (40). Although some foods or supplements used to increase carbohydrate consumption and the macronutrient composition are mentioned in these studies, specifying the amounts of saccharides, starch, or fiber would have provided a deeper insight into the potential differential effects of these dietary items.

Recent studies have emphasized the role of different sugars intake in unpurified high-fat diets. Results from a study of isoenergetic sugar-free high-fat diet (30% fat, 0% sugar, 50% carbohydrates other than sugar) and high-fructose high-fat diet (30% fat, 40% fructose, 10% carbohydrates other than fructose) in rats indicate that fructose is the bioactive component inducing LR (41). Removal of fructose from high-fat diets can even reverse LR and hyperleptinemia, suggesting a causal relationship. Similarly, it has been observed that, compared with rats fed a typical high-fat diet (60% fat, 7% sucrose), LR developed only in those that consumed a significant proportion of their energy intake in the form of liquid sucrose (4% fat, 30% sucrose solution) (43). In a study of healthy humans, 4-week high-fructose feeding (high-fat diets at baseline followed by isocaloric high-fat diets with the addition of 1.5 g fructose per kg body weight per day) led to a continuous rise in fasting plasma leptin concentrations (42).

Fructose consumption may also influence gastric leptin secretion and lead to pathogenic mechanisms that exacerbate LR. A study that evaluated the effects of luminal leptin (mimicking gastric leptin) on the activity and expression of GLUT-2 and GLUT-5 transporters also investigated the role of fructose intake in the modulation of gastric leptin in mice (17). Compared with controls, mice with oral administration of fructose had a significant 10-fold increase in basal leptin levels in gastric juice in 15 minutes. Such effect was independent of changes in plasma leptin levels and not seen after galactose intake. Therefore, although this study did not specifically study the effects of fructose on leptin resistance at the intestinal level, others have suggested the occurrence of peripheral LR to be a function of increased levels of gastric leptin derived from macronutrient intake (33, 50).

The inconsistent results previously discussed indicate a need for further investigation in human studies. Specifying the quality and variation of carbohydrates and the types of dietary fat and further elucidating the molecular mechanisms underlying their associations with leptin sensitivity may help to clarify how dietary intake affects leptin levels and resistance. Further, human studies to specifically analyze the potential effects of dietary fats and carbohydrates (simple and complex) on gastric leptin and LR are necessary to test the hypotheses generated by animal models.

### Protein

Studies examining the relationship between dietary protein and circulating leptin levels have shown an inverse correlation among animal models. One study found that among rats fed a low-protein diet (6% of caloric intake *vs*. 17%), serum leptin levels increased by 100% after 15 days of feeding (44). Total food intake, triglycerides, and weight gain were also higher among the low-protein diet rats than among the control rats. Similarly, Du and colleagues found that rats fed 5% and 8% protein diets had significantly higher leptin levels compared to rats fed 20% protein. However, leptin levels among rats fed 10% or 15% protein did not differ from the 20% protein control group (45).

Few studies on dietary protein in relation to leptin levels have been conducted in humans. One trial conducted among healthy individuals found that an isocaloric high-protein diet did not alter serum leptin levels but increased self-reported satiety. Further, an *ad libitum* high-protein diet resulted in decreased circulating leptin levels and decreased caloric intake, body weight, and fat mass (46).

## Leptin-lowering Effects of Nutritional Interventions to Normalize Leptin Sensitivity

Normalization of leptin sensitivity has been proposed as an efficient strategy to reinforce weight loss and avoid weight regain in treatments for obesity (51). Leptin sensitivity restoration has been described in animal models due to decrements in adipose tissue and leptinemia in conjunction (8, 52). Although leptin is a central component of metabolic adaptations that occur as a function of weight loss and may trigger the restoration of body weight (53), it may be counterintuitive to think that lowering leptin levels will improve weight loss. However, according to the evidence-based model proposed by Zhao S et al. (7), a reduction of bioactive leptin levels in obesity settings may give rise to central and peripheric improvements of leptin sensitization and leptin action.

Therefore, the adiposity- and leptin-lowering effects of dietary interventions, such as energy restriction and fasting could help to recover the sensitivity to this hormone by modulating biological mechanisms involved in LR (8). Given this evidence, several studies have focused on evaluating the effect of restricted-energy diets on leptin levels in humans both in the short and long term (**Table 2**).

**Table 2:** Summary of studies linking nutritional interventions to decrements in leptin concentration*.

Study	Study Characteristics	Main intervention	Main results^a^
Boden G, et al. (54)	- 5 individuals (age 42.2 ± 5.8 y, 2 men and 3 women) with BMI <28 kg.m^2^. - 5 individuals (age 35.6 y ± 6.7 y, 2 men and 3 women) with BMI >28 kg.m^2^.	- Total fast (0 kcal, 52 hours).	- Serum leptin decreased by 64% in individuals with BMI <28 kg.m^2^. - Serum leptin decreased by 72% in individuals with BMI >28 kg.m^2^.
Mars M, et al. (55)	- 34 healthy men (age 23 ± 3 y) with average BMI 22.3 ± 1.6 kg.m^2^.	- 2 days, 62% energy restriction. - With 55% energy from carbohydrates, 20% fat, and 25% protein. - Food ad libitum after intervention.	- Leptin concentration decreased by 27.2%. - Decrements in leptin concentration: not associated with changes in body weight (*r*=0.20, *P*>0.05) or insulin concentrations (*r*=0.21, *P*>0.05), but highly associated with fasting leptin concentrations at baseline (*r*=0.89, *P*<0.0001), the proportion of energy restriction (*r*=0.48, *P*<0.01), and baseline body mass index (*r*=0.44, *P*<0.01), as well as weakly associated with baseline body weight (*r*=0.33, *P*=0.05). -Leptin concentration increased by 37.6% on day 5 (uncontrolled diet).
Mars M, et al. (56)	- 44 healthy men (age 43 ± 5 y with BMI 27.3 ± 3.2 kg.m^2^).	- 4 days, 65%-energy-restricted diet. - With 26% energy from protein, fat 19%, and carbohydrates 55%.	- Leptin concentration declined by 39.4%. - Decrement was associated with an increase in fasting hunger (r=0.42, *P*<0.01), desire to eat (r=0.39, *P*<0.05) and total appetite (r=0.38, *P*<0.05). - Higher absolute (not relative) reduction in individuals with obesity (-4.1 ng/ml) or who were overweight (-1.5 ng/ml) than in participants with BMI <25 kg.m^2^ (2.2 ng/ml).
Wisse BE, et al. (57)	- 21 women with obesity (age 41 ± 3 y, weight: 102 ± 4 kg, 48 ± 1% body fat).	- Group 1: total fast. - Group 2: All-protein, very-low-energy diet (≈455 kcal). - Group 3: Low-energy, balanced-deficit diet (50% maintenance energy). - One week of refeeding with the diet of group 3.	- Leptin levels significantly decreased in the 3 groups at week 1 (up to 66%). - These decrements continued through week 4, though less significantly. - There were greater declines in leptin associated with greater energy deficits. - Decrease in leptin in group 3 was significantly lower than in group 1 from weeks 1 to 4 (*P*=0.026). - By the end of week 5 (refeeding), leptin increased significantly (*P*=0.002) in group 1.
Wadden TA, et al. (58)	- 49 women with obesity (age 45.0 ± 9.6 y with BMI 36.4 ± 4.5 kg.m^2^).	- A 40-week weight loss program. - Group 1 (low-calorie diet): 1000 kcal/day (weeks 2–13); 1200 kcal/day, with 12–15% energy from protein, 55–60% carbohydrates, and 25–30% fat (by week 17); 1200–1800 kcal/day (weeks 21–40). - Group 2 (balanced-deficit diet): usual food intake in first week; 1200 kcal/day, with 12–15% energy from protein, 55–60% carbohydrates, and 25–30% fat (weeks 2–20); 1200–1800 kcal/day (weeks 21–40).	- Leptin decreased more at weeks 6 and 10 in group 1 than in group 2 (*P*<0.05). - At week 10, leptin decreased by 53.0 ± 18.2% in group 1 and 33.2 ± 23.3% in group 2. - Caloric restriction accounted for 14% of the variance in leptin changes at week 6; weight loss did not add significantly to this variance. - At week 10, diet (21%) and weight loss accounted for 36% of the variance in leptin changes. - Week 40: Leptin concentration in group 1 decreased by 37.4 ± 25.9% and ≈35% in group 2.
Varkaneh HK et al. (59)	- 12 studies with 17 arms and a total of 495 women and men (intervention = 249, control = 246).	Fasting and energy-restricted diets and their subgroups: - Energy intake limited to ≤50% of normal energy intake. - Energy restriction regiments with >50% of normal energy intake. - Alternate-day fasting. - Intermittent fasting.	- Overall, leptin levels significantly declined (WMD: -3.69 ng/ml, 95% CI: -5.19, -2.19, *P ≤* 0.001). - Energy-restricted diets only: WMD = -5.23 ng/ml, 95% CI: -7.96, -2.50. - Fasting regimens only: WMD = -2.56 ng/ml, 95% CI: -4.86, -0.25. - Energy intake limited to ≤50% of the normal required daily energy intake: WMD = -4.20 ng/ml, 95% CI: -7.28, -1.12. - Alternate day fasting: WMD = -14.37 ng/ml, 95% CI: -24.03, -4.72. - No statistically significant changes associated with energy restriction regiments with >50% of normal energy intake and intermittent fasting interventions. - Interventions lasting ≥12 weeks: WMD: = -6.92 ng/ml, 95% CI: -10.46, -3.37. - Interventions lasting <12 weeks: WMD = -2.05 ng/ml, 95% CI: -3.54, -0.55. - BMI ≥25 kg.m^2^ at baseline: WMD: = -8.11 ng/ml, 95% CI: -11.97. - BMI <25 kg.m^2^ at baseline: WMD: -1.35 ng/ml, 95% CI: -2.36, -0.35.

*Lowering leptin levels has been proposed as a potential intervention to normalize the sensitivity of this hormone. Studies focused on fasting and energy-restricted diets.

^a^Statistical measures presented in this table are heterogeneous since they are shown as they were available in the reviewed studies.

BMI, body mass index; WMD, weighted average mean.

### Short-Term Interventions

Short-term energy restriction seems to have a leptin-lowering effect. Leptin concentration can be reduced short term through reductions in insulin levels triggered by the low availability of glucose in the bloodstream induced by energy restriction (54, 60). A decline of 72% in serum leptin concentration has been observed in individuals with obesity after an acute exposure to total fast (0 kcal, 52 hours) (54).

A study that evaluated the effect of an energetic restriction of 62% for 2 days found that fasting leptin levels of 34 young, healthy men decreased by 27.2% post-intervention regardless of decrements in body weight. On average, leptin concentration increased by 37.6% on day 5, after returning to an uncontrolled diet (55). Consistently, Mars et al. found that, after a 4-day 65% energy-restricted diet, fasting leptin concentrations of 44 healthy adult men declined by ≈40% (95% CI: -43.6, -34.9%). In absolute terms, this reduction was higher in individuals with obesity or who were overweight than in participants with BMI <25 kg.m^2^. Leptin concentration was not evaluated at the end of this intervention (56).

### Long-Term Interventions

Interventions over longer periods (≈4 – 40 weeks) have suggested that both energy restriction and weight loss can influence reductions in leptin levels and LR. The effect of three types of energy-restricted diets on the leptin concentration of 21 women with obesity was assessed during 4 weeks: 1) total fast, 2) ≈455 kcal in total, mainly from protein, and 3) 50% maintenance energy (57). Leptin levels significantly decreased in all 3 groups at week 1 (up to 66%) and then gradually until week 4 (up to ≈80%). The changes in energy intake in the first week had a greater influence on leptin concentrations than changes in body fat mass. Wadden et al. studied the leptin-lowering effect of a 40-week weight loss program in 49 women with obesity comparing two diets: 1) 1000 kcal/day and 2) 1200 kcal/day. Leptin concentrations were lower in both groups at week 40, but more accentuated in group one, especially at weeks 6 (-55.8%) and 10 (-53%). Leptin concentration in women of the first group decreased by 37.4% at week 40. Long-term changes in leptin were more related to changes in weight than to energy restriction (58).

Both obesity and chronic hyperleptinemia are necessary to cause LR in DIO models (47), thereby suggesting that leptin sensitivity restoration must be achieved by addressing both of these conditions. The literature previously discussed suggests that short-duration energy-restricted diets may lead to fast decrements in leptin levels regardless of changes in adiposity. This effect could be translated into the reversion of leptin resistance which is followed by a more accentuated reduction of leptin concentration triggered by food intake restriction and loss of body fat mass in the long term.

## Discussion

### Contributions and Limitations of Current Evidence and Future Research

One limitation in LR research is that there is no single, universally accepted definition of LR (29). Although circulating leptin levels and leptin content in gastric juice, in conjunction with other variables, are commonly used as proxy measures of low sensitivity, these are not always indicative of LR. Though animal studies allow researchers to evaluate LR, these involve invasive procedures, and they may not perfectly represent the association between nutrients and leptin sensitivity in humans. Therefore, clinical studies are required to standardize reliable tools for diagnosis and studies on leptin sensitivity, just as the glucose clamp techniques were developed to measure insulin sensitivity (61).

Evidence has shown that diet components are linked to the impairment of the leptin system. Diets high in fat, carbohydrates, and specific sugars, such as fructose and sucrose, as well as low in protein are associated with markers of LR, including those of this condition in the periphery. However, studies examining carbohydrates and leptin levels lack sufficient sample sizes. In the future, a nested study within a large-scale epidemiologic trial measuring baseline *vs*. follow-up blood samples for serum leptin levels and other LR indicators could help to better elucidate this complex relationship. Further, rigorous studies focused on specific diet composition and emphasizing the role of substituting certain nutrients with others would be instrumental in further advancing the understanding of the relationship nutrition-leptin system in humans. Given that the pathophysiological processes involved in obesity, the leptin system, and diet are highly complex, nutritional metabolomics studies would be helpful to evaluate the role of dietary patterns in LR through the identification of their metabolic signatures, adherence, and metabolic responses as it has been done to study other diet-disease relationships (62).

Multiple studies have evaluated the influence of nutritional interventions on leptin concentration as a strategy to restore sensitivity to this hormone. A 2020 systematic review and meta-analysis summarized 12 controlled clinical trials published between 2009 and 2018 to evaluate the effect of dietary approaches on leptinemia. Including data from 495 individuals in multiple countries, this review showed that a significant decrease in serum leptin levels was associated with fasting and energy-restricted diets (weighted mean difference: -3.69 ng/ml, 95% CI: -5.19, -2.19, *P* ≤ 0.001) (59). However, the authors found a high degree of heterogeneity regarding dietary intervention procedures, duration, and variation of participant characteristics (e.g., adults classified with and without obesity) in the analyzed studies, making generalization of results difficult. This review also documented important patterns regarding dietary protocols that can be relevant to future interventions (59). For example, highly restrictive diets seem to have a more relevant leptin-lowering effect than moderate energy restriction (≤50% *vs*. >50% of required daily intake), and energy-restricted diets are better than fasting to reduce leptin concentrations. In line with the present review, the previous work suggests that the evidence regarding the role of nutritional interventions in leptin sensitivity normalization is still inconclusive. 

As previously mentioned, leptin concentration is a function of body fat and food availability (4, 6). Hence, interventions specifically directed to reduce weight by lowering caloric intake may also impact leptin sensitivity (63, 64) through a “positive feedback loop”. That is, an initial intake reduction (and/or higher physical activity levels) (65) followed by weight loss, decreases leptin concentration and sensitivity improvements that lead to reduced intake. This mechanism suggests that weight loss is a key component to decrease leptin concentrations in dietary interventions, but also that it has a greater long-term influence by accentuating decrements in this hormone triggered by energy restriction in shorter periods. However, these benefits offered by diet modifications and weight control may be in part hampered by leptin-related physiological adaptations to weight loss as well as by behavioral constrains, including a decreased calorie expenditure, increased appetite, and diet noncompliance as interventions progress (66–69).

### Clinical and Public Health Translations

The translation of nutrition interventions into recommendations at the population level can be challenging. For example, very-low-energy regimens for long periods could create a high psychological burden in individuals if they are not monitored and incentivized by well-trained health professionals. However, once the relationship between the leptin system and diet is better understood and the potential barriers and facilitators have been evaluated through implementation science studies, it is more likely that translation into practice will be feasible in strictly controlled clinical settings.

Though nutrition-based interventions for reversing LR were the main interest of this review, pharmacological studies are also being explored. Animal studies are investigating if drugs can restore leptin sensitivity and normal leptin levels among mice with DIO, predominantly focusing on compounds that act as leptin sensitizers (70). For example, Withaferin A, a naturally occurring compound with an mRNA profile like known leptin sensitizers, was investigated as a treatment to reverse LR and reduce body weight. It reduced murine body weight by 20-25% and reversed other metabolic abnormalities, such as hepatic steatosis (71). Though these treatments have been effective in mice, they have not yet been shown to be safe for human use (70, 71). Leptin neutralizing antibodies are another therapeutic intervention against LR studied in mice (28). Under obesity conditions, the pharmacological capability of these compounds to achieve a “partial” reduction of bioactive leptin levels has shown crucial benefits, including an enhanced leptin sensitivity in the central nervous system and a higher degree of peripheral leptin sensitivity (28). Once the safety of these pharmacological strategies has been determined in humans, they could contribute to counteracting the leptin-related metabolic adaptations (decreased calorie expenditure and increased appetite) seen in weight-loss interventions.

Researchers have begun to disentangle how genetic and environmental factors, including diet, interact to increase the risk of obesity (72, 73), but the complexity of this relationship will most likely take many years to fully elucidate. Research on polymorphisms in the genes for leptin and other energy-regulating hormones like ghrelin and insulin raises the possibility of precision nutrition approaches, such that dietary guidance may be customized to an individual’s specific genetic makeup (74). However, precision nutrition research will require a better understanding of the gene–phenotype relationship and the molecular mechanisms through which leptin and other hormones exert their downstream effects on physiological systems. More evidence on how phenotypes are expressed in the interaction between genes and diet will also be instrumental in accounting for potential scenarios where specific dietary patterns can mask underlying genetic mutations relevant to the leptin system.

### Conclusion

Recent advances in understanding the interconnection between diet and the leptin system hold promise in providing better treatments for individuals with DIO. The link between high-fat and low-protein diets, the overconsumption of carbohydrates, fructose, and sucrose, and biomarkers of impaired leptin action, as well as recent research demonstrating that this condition can be reversed, highlights the possibility of utilizing nutritional strategies to tackle LR. Nevertheless, a harmonized definition of LR and the development of reliable methods to measure this condition in humans, as well as large-scale, translational, and clinical research involving rigorous nutrition science methods are needed before this promising approach can be implemented at the population level.

## Author Contributions

KMH, AAF, and MM conceptualized the manuscript and performed the literature search and manuscript drafting. JM, SNB, AM, and MT supervised and revised the manuscript. All authors contributed to the article and approved the submitted version.

## Funding

This project was supported by scholarships from the Mexican Council of Science and Technology (Spanish acronym: CONACYT), Fundación México en Harvard, and Harvard University provided to KMH. Additionally, AAF received support from the NIH Training Grant in Academic Nutrition #2T32DK007703-26 which facilitated carrying out this project.

## Conflict of Interest

The authors declare that the research was conducted in the absence of any commercial or financial relationships that could be construed as a potential conflict of interest.

The reviewer ZT declared a shared affiliation, with no collaboration, with the authors to the handling editor at the time of review.

## Publisher’s Note

All claims expressed in this article are solely those of the authors and do not necessarily represent those of their affiliated organizations, or those of the publisher, the editors and the reviewers. Any product that may be evaluated in this article, or claim that may be made by its manufacturer, is not guaranteed or endorsed by the publisher.
